# Comparative chromosomal analysis and evolutionary considerations concerning two species of genus *Tatia* (Siluriformes, Auchenipteridae)

**DOI:** 10.3897/CompCytogen.v7i1.4368

**Published:** 2013-04-10

**Authors:** Roberto Laridondo Lui, Daniel Rodrigues Blanco, Vladimir Pavan Margarido, Waldo Pinheiro Troy, Orlando Moreira Filho

**Affiliations:** 1 Departamento de Genética e Evolução, Universidade Federal de São Carlos, Rodovia Washington Luís (SP 310) Km 235, CEP: 13565-905, São Carlos, SP, Brazil; 2 Centro de Ciências Biológicas e da Saúde, Universidade Estadual do Oeste do Paraná, Rua Universitária 2069, CEP: 85819-110, Cascavel, PR, Brazil; 3 Departamento de Ciências Biológicas, Universidade Estadual de Mato Grosso, Rodovia MT 358, Km 07, s/n. Jardim Aeroporto, CEP: 78300-000, Tangará da Serra, MT, Brazil

**Keywords:** Pericentric inversions, NORs, C-banding, 5S rDNA-FISH, 18S rDNA-FISH

## Abstract

Auchenipteridae is divided in two subfamilies, Centromochlinae and Auchenipterinae. Centromochlinae has 31 valid species, from which 13 are included in the genus *Tatia* Miranda Ribeiro, 1911. Among these, *Tatia jaracatia* Pavanelli & Bifi, 2009 and *Tatia neivai* (Ihering, 1930) are the only two representative species from the Paraná-Paraguay basins. This study aimed to analyze cytogenetically these two species and thus provide the first chromosomal data for the genus. Although *Tatia jaracatia* and *Tatia neivai* presented 2n=58 chromosomes, some differences were observed in the karyotypic formula. The heterochromatin was dispersed in the centromeric and terminal regions of most chromosomes of *Tatia jaracatia*, and only in the terminal region of most chromosomes of *Tatia neivai*. The AgNORs were detected in the subtelocentric pair 28 for both species, which was confirmed by FISH with 18S rDNA probe. The 5S rDNA sites were detected in four chromosome pairs in *Tatia jaracatia* and three chromosome pairs in *Tatia neivai*. Both species of *Tatia* presented great chromosomal similarities among themselves; however, when compared to other species of Auchenipteridae, it was possible to identify some differences in the karyotype macrostructure, in the heterochromatin distribution pattern and in the number and position of 5S rDNA sites, which until now seems to be intrinsic to the genus *Tatia*.

## Introduction

Among the Siluriformes, Auchenipteridae comprises a fish group endemic to the Neotropical region. The family comprises 20 genera and about 90 species (Ferraris Jr 2007), 74 of which have already been registered in Brazil (Akama and Sarmento-Soares 2007). According to Ferraris Jr (2003), Auchenipteridae is subdivided in two subfamilies, Centromochlinae and Auchenipterinae, which form monophyletic groups (Birindelli 2010). Most of the Auchenipteridae genera belong to the subfamily Auchenipterinae, with only *Centromochlus* Kner, 1857, *Gelanoglanis* Böhlke, 1980, *Tatia* Miranda Ribeiro, 1911 and *Glanidium* Lütken, 1874 allocated in Centromochlinae (Soares-Porto 1998). The subfamily Centromochlinae has 31 valid species (Ferraris Jr 2007), and in a revision of the genus, 12 species are described of *Tatia* (Sarmento-Soares and Martins-Pinheiro 2008). After this revision, a new species was described for this genus, *Tatia jaracatia* Pavanelli et Bifi, 2009, which is endemic to the Iguaçu River, a tributary of the Paraná River basin (Pavanelli and Bifi 2009).

The genus *Tatia* is found in the eastern region of the Andes, with wide distribution in South American drainages (Sarmento-Soares and Martins-Pinheiro 2008). Generally, fishes from this group are found in lentic environments of streams, rivers and lagoons and have nocturnal habits (Lowe-McConnell 1987). Most species can be found in rivers belonging to the Amazon River basin (Sarmento-Soares and Martins-Pinhiero 2008). The species studied in this paper [*Tatia neivai* (Ihering, 1930) and *Tatia jaracatia*]represent the only two species from the Paraná-Paraguay basins that belong to the genus *Tatia*, being *Tatia neivai* widely distributed in the Paraná and Paraguay basins and absent in the Iguaçu River basin.

Chromosomal analyses in Auchenipteridae are scarce and restricted to few species of the genera *Ageneiosus* La Cepède, 1803, *Auchenipterus* Bleeker, 1862, *Glanidium* and *Parauchenipterus* (Linnaeus, 1766). The two analyzed *Ageneiosus* species demonstrate diploid number of 56 chromosomes (Fenocchio and Bertollo 1992), while the other analyzed species [*Glanidium ribeiroi* Haseman, 1911, *Parauchenipterus galeatus* (Linnaeus, 1766) and *Auchenipterus osteomystax* (Miranda Ribeiro, 1918) cited as *Auchenipterus nuchalis* (Spix et Agassiz, 1829)] have 58 chromosomes (Fenocchio and Bertollo 1992, Ravedutti and Júlio Jr 2001, Fenocchio et al. 2008, Lui et al. 2009, Lui et al. 2010). Until now, there were no chromosomal studies in *Tatia* species. Therefore, this study aimed to cytogenetically analyze the two species of the Paraná-Paraguay drainage belonging to the genus *Tatia* (*Tatia neivai* and *Tatia jaracatia*), generate the first chromosomal data concerning the genus and thus allowing differentiation of closely related species.

## Material and methods

Chromosomal analysis was performed on 17 specimens (15 males and 2 females) of *Tatia neivai* from Machado River, a tributary of the Bugres River, Paraguay River basin, Denise city, Mato Grosso, Brazil (14° 40'43"S, 57°00'47"W), and 10 specimens (7 males and 3 females) of *Tatia jaracatia* from the Iguaçu River basin, Capanema city, Paraná, Brazil (25°35'19"S, 53°54'48"W). The specimens were deposited in the fish collection of Museum of Zoology of University of São Paulo (*Tatia jaracatia*, MZUSP 109792; *Tatia neivai*, MZUSP 109794).

Specimens were previously treated with 0.05% colchicine solution (1 ml/100 g body weight), 30-40 minutes before sacrifice, and the cell suspension of mitotic chromosomes was obtained from the anterior kidney cells (Bertollo et al. 1978, Foresti et al. 1993). Thirty metaphase plates from each fish were examined and 10 of the best mitotic metaphases were used to measure karyotypes. Chromosome morphology was determined according to Levan et al. (1964). The fundamental number (NF) was calculated considering metacentric (m), submetacentric (sm) and subtelocentric (st) chromosomes as having two arms, and acrocentric chromosomes (a) as having only one arm. The heterochromatic pattern was determined according to Sumner (1972) with modifications in the staining process (Lui et al. 2012), and the nucleolus organizer regions (NORs) were identified using silver nitrate impregnation (Howell and Black 1980). Both methods were administered sequentially, following the conventional chromosome staining with Giemsa (sequential analysis).

The fluorescence *in situ* hybridization (FISH) was performed according to Pinkel et al. (1986). The 5S and 18S rDNA probes were obtained according to Martins et al. (2000) and Hatanaka and Galetti Jr (2004), respectively. The 5S and 18S rDNA probes were labeled by nick translation with biotin-16-dUTP and digoxigenin-11-dUTP (Roche), respectively. Probes labeled with biotin were detected and amplified with avidin-FITC and anti-avidin-biotin (Sigma). The other probes labeled with digoxigenin were detected with anti-digoxigenin-rhodamine (Roche). Chromosomes were counterstained with DAPI solution and analyzed in the epifluorescence microscope Olympus BX50. Images were captured with the DP2-BSW software (Olympus).

## Results

### Tatia jaracatia

**Figure 1.**
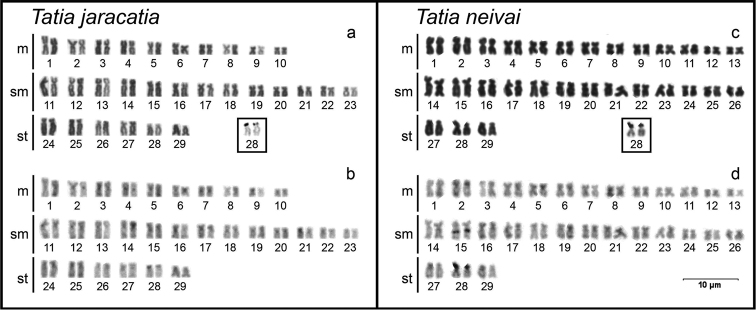
Karyotypes of *Tatia jaracatia* (**a, b**) and *Tatia neivai* (**c, d**) stained with Giemsa (**a, c**) and sequentially C-banded (**b, d**). The AgNORs bearing chromosomal pair is presented in box.

**Figure 2.**
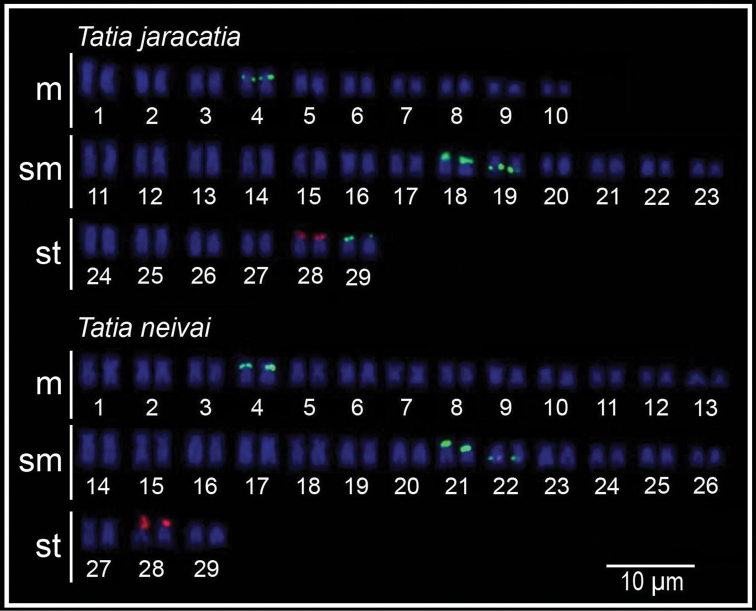
Karyotypes of *Tatia jaracatia* and *Tatia neivai* 5S rDNA-FISH (FITC, green) and 18S rDNA-FISH (digoxigenin, red).

Cytogenetical analysis revealed the diploid number of 58 chromosomes (20m+26sm+12st, FN=116) (Fig. 1a). The heterochromatin presented itself disperses in the centromeric and terminal regions of most chromosomes of the karyotype (Fig. 1b). The silver nitrate impregnation showed only the subtelocentric pair 28 marked in the terminal position of the short arm (Fig. 1a, in box). FISH with 18S rDNA probe showed only one labeled chromosome pair (pair 28) corresponding to the silver nitrate impregnation. The 5S rDNA sites were detected in 4 chromosome pairs (pairs 4, 18, 19 and 29), on the short arm in interstitial position of the metacentric pair 4, on the short arm in terminal position of the submetacentric pairs 18 and 29, and on the long arm in interstitial position of the submetacentric pair 19 (Fig. 2).

### Tatia neivai

Cytogenetical analysis revealed the diploid number of 58 chromosomes (26m+26sm+6st, FN=116) (Fig. 1c). The heterochromatin showed itself poorly marked and dispersed in the terminal region of most chromosomes of the karyotype, with the exception of two conspicuous blocks: one in interstitial position on the long arm of submetacentric pair 15, and other in terminal position on the short arm of subtelocentric pair 28 (Fig. 1d), corresponding to the NORs (Fig. 1c, in box). FISH with 18S rDNA probe showed only one labeled chromosome pair, the subtelocentric pair 28, corresponding with the silver nitrate impregnation. The 5S rDNA sites were detected in 3 chromosome pairs (pairs 4, 21 and 22), being in the interstitial position of the short arm of metacentric pair 4, in terminal position of the short arm of submetacentric pair 21, and in interstitial position of the long arm of submetacentric pair 22 (Fig. 2).

No intraspecific polymorphism related to diploid number, karyotypic formula, C banding, 5S and 18S rDNA (including AgNORs) were observed in both species.

## Discussion

Chromosomal studies in Auchenipteridae have shown that most analyzed species have diploid number of 58 chromosomes (Ravedutti and Júlio Jr 2001, Fenocchio et al. 2008, Lui et al. 2009, Lui et al. 2010), with the exception of species from the *Ageneiosus* genus that have 56 chromosomes (Fenocchio and Bertollo 1992). The genus *Tatia* is included in the subfamily Centromochlinae, which had only one species with chromosomal analysis to date, *Glanidium ribeiroi*, which also has 2n=58 chromosomes, as well as the two species of *Tatia* analyzed in this paper. The 2n=58 chromosomes is shared by species of the *Auchenipterus* and *Parauchenipterus* genera (subfamily Auchenipterinae), and 14 out of the 16 already analyzed species of the Doradidae family (Eler et al. 2007, Milhomem et al. 2008), which is considered sister-group of Auchenipteridae (Pinna 1998). Thus, it is likely that 2n=58 chromosomes is basal, not only in Auchenipteridae, but also in Centromochlinae. This hypothesis is reinforced by the fact that 2n=58 chromosomes is considered basal for Doradidae (Milhomem et al. 2008).

The fundamental number (FN=116) found for the two *Tatia* species in this paper is higher than found in other Auchenipteridae species studied so far. This difference is due to an increase in the number of chromosomes bearing two arms in the detriment of chromosomes carrying only one arm (Fig. 1a, c). This absence of acrocentric chromosomes was not detected in other species of the family yet, and seems to be an intrinsic characteristic of the genus *Tatia*, or at least of a specific clade formed by the species studied here. Thus, considering the maintenance of the diploid number, the variations in the karyotypic formula and FN of analyzed species, when compared with other species from others Auchenipteridae genus, it is evident that non-Robertsonian rearrangements, here represented by pericentric inversions, must be active mechanisms in the karyotypic diversification of *Tatia* species.

The heterochromatin distribution pattern found in *Tatia jaracatia* and *Tatia neivai* differs in some aspects from other Auchenipteridae species. Besides heterochromatic blocks in the terminal region of chromosomes, which are commonly found in most Auchenipteridae species, heterochromatin sites were observed in the centromeric region of some chromosomes in *Tatia jaracatia*, and a conspicuous block in the interstitial region of the submetacentric pair 15 of *Tatia neivai* (Fig. 1b, d). No interstitial heterochromatin blocks were detected in *Tatia jaracatia*.

The silver nitrate impregnation had only one subtelocentric chromosome pair marked on the short arm in terminal position (pair 28) in both species (Fig. 1, in box), as confirmed by FISH with 18S rDNA probe (Fig. 2). This pair is likely correspondent between species. According to Ravedutti and Júlio Jr (2001), simple NORs in interstitial position seem to be a characteristic of Auchenipteridae. In Doradidae (sister-group), NORs vary in number and type of the bearing chromosome pairs among the 16 species studied so far (Milhomem et al. 2008). According to the same authors, pericentric and paracentric inversions may have acted in the karyotype evolution of the group changing the location of these sites. A similar situation may have occurred in Auchenipteridae. Although there are slight variations in the location of these sites among the species of this family, it is likely that the 18S rDNA bearing chromosome pairs are corresponding among them. Given this context, the fact that the NORs are always located on a single chromosome pair may suggest this condition is a putative basal character of the clade composed by the Auchenipteridae and Doradidae families. Regarding the *Tatia* species, the data for this chromosome pair also suggest a conserved status for the genus because of the location and type of the chromosome pair bearing these genes.

The data of 5S rDNA sites physical mapping by FISH in Auchenipteridae are scarce and only refer to *Parauchenipterus galeatus* populations (Lui et al. 2010), which presented sites located in interstitial position of two submetacentric pairs: one pair on the short arm and another on the long arm, which change in location in the karyotype among populations of this species. In both species of *Tatia*, two submetacentric pairs (pairs 18 and 19 in *Tatia jaracatia*; pairs 21 and 22 in *Tatia neivai*) bearing the 5S rDNA cistrons were observed, with location similar to the two 5S rDNA bearing chromosome pairs in the different populations of *Parauchenipterus galeatus* (Lui et al. 2010). It is likely that due to the similar morphology and location, these pairs may be considered correspondent among the species, even though they present great phylogenetic distance within the family. The metacentric pair 4 shows 5S rDNA sites in interstitial position in the short arm, which is shared by both *Tatia* species. The 5S rDNA cistrons of pair 4 and the other two aforementioned can be considered as matching between *Tatia jaracatia* and *Tatia neivai*; however, the site present in terminal position on the short arm of the subtelocentric pair 29 seems to be a unique feature of *Tatia jaracatia*. Despite the multiple conditions with more than two pairs bearing the 5S rDNA cistrons being shared by many species of Auchenipteridae, this marker appears to present greater diversity in the family Auchenipteridae when compared to other commonly used markers.

According to the phylogeny of Soares-Porto (1998), *Tatia neivai* is sister-group of *Tatia bohemia* Koch et Reis, 1996, being the latter found only in the Uruguay River, and with *Tatia jaracatia*, these three species are the only valid species for the La Plata basin (Uruguay, Paraguay and Paraná Rivers). Thus, despite the great geographic distance that separates the species analyzed in this paper, it is possible to assume that *Tatia neivai* and *Tatia jaracatia* present significant phylogenetic proximity, which explains the great similarity found with most of the markers. However, when comparing both *Tatia* species from this paper with other species of Auchenipteridae, we can observe that the karyotypic formula (mainly due to the lack of acrocentric chromosomes) and the distribution pattern of the heterochromatin and 5S rDNA sites differ from the rest of the group showing some characteristics which until now appear to be intrinsic to the genus *Tatia*.
